# Exploration on the Core Elements of Value Co-creation Driven by AI—Measurement of Consumer Cognitive Attitude Based on Q-Methodology

**DOI:** 10.3389/fpsyg.2022.791167

**Published:** 2022-04-01

**Authors:** Yi Zhu, Peng Wang, Wenjie Duan

**Affiliations:** ^1^Business School, Shanghai Jianqiao University, Shanghai, China; ^2^International Governance Research Center for Cyberspace, Fudan University, Shanghai, China; ^3^Faculty of Economics & Management, University of Putra, Kuala Lumpur, Malaysia; ^4^Social and Public Administration School, East China University of Science and Technology, Shanghai, China

**Keywords:** artificial intelligence (AI), value co-creation (VCC), Q-methodology, cognitive attitude, key factors

## Abstract

Value co-creation (VCC) goes through the stage of co-production, customer experience, service-dominant logic, and service ecosystem. The integration of science and technology has become a key factor to the process of VCC. The rise and application of artificial intelligence (AI) technology has added a new driving force to VCC and began to affect its original practical logic. Based on the consumer perspective, this study uses Q-methodology to measure consumer cognitive attitude toward the use of AI technology in VCC, aiming to explore the key factors that affect VCC. The study found that content quality, information security, efficiency concern, and degree of manipulation have become the important concerns of consumers for VCC under AI integration. Moreover, their different statements have their specific focus and direction. The study demonstrates and analyzes the importance of the four factors and proposes the combination of human and non-human actors (technology and system) to shape the new model of VCC in the future, which is worth further deepening and exploring for academia and enterprises.

## Introduction

As a new kind of value creation method, the academic circle has gradually paid attention to value co-creation (VCC) since Prahalad proposed it. Now, VCC has become the focus of current marketing theory and practice research (Prahalad and Ramaswamy, 2000). The most representative is the service leading logic theory [service-dominant logic (S-D)] proposed by Vargo and Lusch (2004), who noted that consumers are no longer the external resources of enterprises, but the co-creators of value. Thus, the goods-dominant logic of commodity exchange emphasized in the previous stage of commodity dominance is overthrown. In addition, consumers and business subjects are closely linked together to jointly carry out a series of interactive and innovative activities for commodities and services to realize the common value expectation (Payne et al., 2008). Then, Vargo and Lusch (2010) further extended the concept of service ecosystem based on the dominant basis of S-D, focusing on multi-level network and dynamic ecosystem and emphasizing diversified stakeholder involvement. Domestic scholars, such as Guo Chaoyang and Li Lei, think it is the new direction of service leading logic (Chaoyang et al., 2012; Lei et al., 2013). Zhaoquan et al. (2016) made a thorough combing from the perspective of consumer experience to service ecosystem, emphasizing the evolution trend of service ecosystem in VCC research.

In recent years, with the maturity and application of artificial intelligence (AI) technology, AI has been embedded in the daily “business-consumer” VCC process, which has changed the traditional interaction model and emerged a series of new co-creation practice forms (Erdem et al., 2016; Jiaxun, 2016). The development of digital technology and the emergence of social media and communities have provided a broad space for the practice of AI, promoted more extensive dissemination, diversified interaction, and intelligent application, and thus profoundly affected the process, form, and performance of VCC (Singaraju et al., 2016). Driven by AI, new content and management paradigms have emerged for VCC (Kao et al., 2016; Ramaswamy and Ozcan, 2016). Re-understanding and recognizing VCC driven by new technology are increasingly important and urgent, which will play a vital guidance and reference role for the practice of enterprises, consumers, and relevant stakeholders. Customers engage in VCC when they have positive attitudes toward VCC during interactions with service providers. This tendency of customers to engage in interaction and dialogue with service providers is referred to as a customer’s VCC attitude (Shamim et al., 2016). In the literature related to VCC, the research involving non-human behavior elements (technology, system, and others) is still in the initial stage. Only Hao et al. (2021) analyzed the importance of non-human behavior in VCC by using an actor network (ANT). Based on the evolution logic of VCC and combined with the practical application of AI technology in the process of VCC, the present study attempts to explore consumers’ cognition and expectation of AI technology and VCC and clarify the core elements of VCC driven by new technology.

This research aims to explore consumers’ acceptance and attitude toward the use of artificial intelligence technology in value co-creation based on the intervention of artificial intelligence and the specific application of artificial intelligence between consumers and enterprises. At the same time, the Q classification method is used to analyze the classification of consumer-related attitudes to analyze the role of artificial intelligence in value co-creation, thereby helping enterprises, industries, and consumers better promote the implementation of value co-creation.

## Literature Review

Value co-creation has gone through the stage of co-production (Vargo et al., 2008), customer experience, S-D (Heinonen et al., 2010), and service ecosystem (Pinho et al., 2014). Each stage shows its own characteristics and connects the evolution logic of VCC in the core dimension.

Co-production is a form of VCC in the production stage, which is dominated by objective resources. Enterprises lead the creation of value, whereas consumers participate in the co-production of goods and services as potential resources and co-producers. Through the interaction between enterprises and consumers, more value can be brought to enterprises and consumers (Wikstrom, 1996). In this context, the core point of VCC lies in that the enterprise is the leader of VCC, whereas the consumer is a kind of productive resource (Ramiìrez, 1999). In the goods-dominant logic, companies produce goods or services that they exchange for money with customers. The emphasis is on the linear, transactional exchange between two or more entities in the market. In SDL, the demarcation between customers and suppliers becomes obsolete as this logic does not differentiate between “givers” and “takers.” Instead, all actors integrate resources. Terms, such as “customer” and “supplier,” imply an exchange relationship that, according to SDL, is static and does not reflect economic reality (Hartwig et al., 2021). A two-way interaction has become a form of communication between enterprises and consumers that breaks through the tradition (Ojasalo, 2010). Enterprises began to pay attention and incorporate the opinions and feedback of the consumer. The process of value creation takes place in each stage of the whole co-creation and keeps pace with value creation (Payne et al., 2009).

Consumer experience is a further evolution based on co-production. The VCC model shifts from the enterprise as the center to the consumer experience as the center, emphasizing the important contribution of consumers to value creation (Liangjie et al., 2017). Competition among enterprises is becoming increasingly fierce. Consumers pay increasing attention to their own personalized experience. Therefore, customer experience has become the basis for co-creation between enterprises and consumers, which plays an important role for enterprises to compete (Reynoso, 2021). Enterprises and consumers jointly create a consumption experience and run through the whole process of VCC. Constantly improving and optimizing the experience environment has become an important embodiment of value creation (Lengnick-Hall, 1996). At this stage, the core of VCC lies in that enterprises and consumers have become the important subjects of VCC (Prahalad and Ramaswamy, 2004). Continued interaction and dialogue are going on throughout the co-creation process (Ramaswamy and Ozcan, 2013). Consumer experience has become the basis and core advantage of VCC (Ramaswamy and Chopra, 2014).

Service-dominant logic is the replacement of commodity-led logic. Vargo and Lusch (2011) unified products and services and believed that all economies are service economies, in which consumers participate in relational exchange and co-production. Value is determined and produced by consumers. In addition, the proposition of VCC was proposed based on the service-leading logic, which gradually increased from the initial 9 FP to 11 items, covering the dimensions of value proposition, operational resources, integrator, distribution mechanism, and others. The core elements of VCC were systematically redefined. On this basis, Grönroos and Voima (2013) further divided service dominance into consumer and supplier service logic, emphasizing that consumers are value creators and suppliers are value facilitators, thereby enriching the participants further. Service is the use of knowledge and skills to produce real value (value-in-use) from the potential values of products and is SDL through the collaboration of multiple actors and resource integration to create value for the benefit of all (Siddique et al., 2021). According to SDL, the firm is a value facilitator, which embeds a potential value in goods and services during the planning, design, production, and delivery phases. Customers are value creators who create value during the consumption process, such as value-in-use (Shamim et al., 2017). The core of VCC in this stage lies as follows: service is embedded in all kinds of economic activities and follows the paradigm of service economy; the consumer becomes the leader of value creation; the subjects participating in VCC are not limited to enterprises and consumers, and more possibilities exist.

With the development of the technology, the form of VCC becomes increasingly complex and ecological, and the perspective of the service ecosystem begins to expand the service dominance (Chandler and Vargo, 2011; Vargo and Lusch, 2016). VCC emphasizes the network link among multiple actors based on Action to Action (A2A) to realize value creation through system and technology according to their own value propositions (Williamson, 2000). From the perspective of the network ecosystem, it is emphasized that all participants play a promoting role in VCC. In a complex, loosely coupled system, value is achieved through service exchange and resource integration (Akaka et al., 2013). In the whole process of value creation, technical elements become increasingly important and become the key to guaranteeing the link of the subject, the achievement of results, and the improvement of efficiency (Payne et al., 2009).

In accordance with the evolution trend of value creation, as a kind of drive element embedded in the whole process of value creation, with its own uniqueness, AI technology links the participation of multiple main bodies and builds a complex relationship network and system. Then, AI technology began to change the original value to create the form and content to understand and analyze the AI technology system. This technology will help to better grasp the scope of VCC.

The research on the use of AI in VCC from January 2015 to August 2021 was selected as the time interval, with the help of the web of science database,. According to the keywords, AI, deep learning, machine learning, neural network, robot, VCC, and others, a total of 68 papers on related topics were selected. Among them, 45 papers have been published in the last three years, illustrating AI and VCC, which have become a research hotspot in recent years and attracted the attention of the academic community. After reviewing all kinds of literature, AI and VCC have three viewpoints. First, AI is a support service provider of VCC. Many studies discussed how intelligent technology can support the realization of VCC, such as the advantages of the neural network, deep learning, and other methods in predicting co-creation behavior and market trends. The support it provides includes other cognitive support for service providers, including evaluating the usefulness of customer reviews (Singh et al., 2017), supporting complex new product development decisions (Thieme et al., 2000), and providing homogeneous segmentation solutions. Boone and Roehm (2002) and Huang and Rust (2018) drew a map showing how companies should make decisions between humans and machines to accomplish mechanical, analytical, intuitive, and empathic tasks and achieve VCC. Second, AI realizes resource integration between the service provider and beneficiary. AI can understand customer needs and preferences to achieve resource integration between service providers and beneficiaries (Glushko and Nomorosa, 2013). By identifying customer needs and preferences, AI can add human-like functions to co-creation behaviors (Fan et al., 2016; Van Doorn et al., 2017) and play a positive role in VCC. Third, AI supports the well-being of beneficiaries. Studies discussed how AI and robots can support VCC for beneficiaries (Marinova et al., 2017). For example, studies identified six roles in the value network of the elderly (enabler, invader, ally, substitute, extended ego, and deactivator) and linked three health support functions of AI, namely, protection, social contact, and cognitive support. Considering the combination of the beneficiary’s existing value network and AI technology, the beneficiary’s experience of VCC is promoted, and individual well-being is further enhanced. Most of the related literature in the past has been based on theoretical elaboration or research on the specific application of specific AI technology for value co-creation. These studies are scattered and unsystematic. Relevant literature scarcely focuses on consumer attitudes toward AI application in value co-creation as a whole, and no systematic attitude research exists.

Emphasizing the AI technology in value to create a positive impact, a substantial amount of literature mentions the risks of AI, mainly from many scholars who believe in the existence of certain information security risks; showing the information collection, processing, storage, analysis process, for the “enterprise-customer” to produce the interaction between the limit; and make the entire interaction insecure (Giebelhausen et al., 2014; Jarek and Mazurek, 2019). Ethical issues are also triggered by AI, focusing on the customer’s right to know in the process of use, anthropomorphic communication ability, among others, and discussing the equality relationship between human and machine in anthropomorphic interaction (Huang and Rust, 2021). In addition, some scholars have discussed the threat of AI technology to humanoid intelligence and service interaction, which further limits the decision-making and thinking of participants in the interaction process. AI will move from an emphasis on analysis, intuition, and empathy to one where it can replace human intelligence, threatening to replace human emotion (Huang and Rust, 2018; Iio et al., 2020).

Earlier studies have discussed the superiority of AI technology, and studies with concerned attitudes also exist. From the perspective of consumers themselves, what is their attitude toward the application of AI in value co-creation? This research hopes to solve and innovate on major issues on the basis of previous research, promote the enrichment of AI-related research on VCC, and explore the real demand and expectation for AI from the perspective of consumers. The specific implementation strategy is improved and innovated from three aspects: research object, influencing factors, and research method. First, the study introduces AI as a new subject and object to expand the breakthrough in the research object. Second, in terms of influencing factors, based on the new practice scenario of AI embedding in VCC, relevant influencing factors are verified, and new discoveries are made. Finally, in the research method, the mixed research method, namely, the Q method, is attempted. From the perspective of consumers themselves, the study explores their demands and expectations for AI in VCC and uses new methods to achieve innovation in research results.

## Materials and Methods

If AI is embedded in the VCC of “enterprise and consumer,” what will consumer cognitive attitude and expectations toward it be? In this study, the Q method is used to measure and observe it, to present consumers’ concerns about the application of AI technology, and its current situation in VCC.

### Research Methods and Strategies

The Q method originates from the British physicist and psychologist William Stephenson, who believes that the Q method is a research method that defines the cognitive attitude with the help of the respondents’ own statements or opinions. The Q method specializes in studying the subjective attitude of human beings (Sallot, 2012). This research method has been applied in many disciplines, such as psychology, sociology, and management. The advantages of this research method are as follows. First, this method can be widely searched for viewpoints, avoiding the subjective bias of researchers. Second, the research process combines qualitative and quantitative research methods, which belong to mixed research, and the advantages of the two kinds of research methods are integrated. Third, the research does not need several samples but can be achieved with a small sample (*N* < 30) (Chan et al., 2011). The whole analysis process is divided into three key links: Q sample establishment, P sample measurement, and Q classification (Thomas and Watson, 2002). For this study, the implementation steps of the Q method were analyzed one by one.

Q methodology is a means of extracting subjective opinion. It has since been applied outside the field of academic psychology, most notably in the fields of communication and political science and more recently in the behavioral and health sciences. It was interested in providing a way to reveal the subjectivity involved in any situation—it is life as lived from the standpoint of the person living it, which is typically passed over by quantitative procedures, and it is subjectivity in this sense that Q methodology is designed to examine.

The instrumental basis of Q methodology is the Q sort technique which conventionally involves the rank-ordering of a set of statements from agree to disagree. It requires the participant to evaluate (or sort) a number of items along a continuum from, for example, “very like me” to “very unlike me.” The respondent arranges the statements into a forced normal distribution of most to least agreement, yielding a model of subjective preferences within the given “universe of discourse.” The data from Q methodology are literally what participants make of a pool of items germane to the topic of concern when asked to rank them.

The basic steps of the Q method are as follows: (1) collect opinions and opinions related to the propositions; (2) extract evaluation factors; (3) select subjects with significant differences; (4) carry out Q sorting, forcing subjects to rank Q propositions according to a certain number; and (5) analyze and interpret according to the classification results, select some declarative sentences and ask the subjects to explain, and analyze the results in combination with the interviews after data analysis. The specific operation process of this research is as follows:

First is the Q sample establishment stage. The so-called Q sample refers to a series of subjective statements (topic propositions) for the tester to test. These statements are related to the research topic, and they are obtained and refined through interviews and inquiries. Here, for consumers to create value in the application of AI technology; the selection of the scholars; and the general consumer, technical staff, and managers (marketing department), a total of 56 people were asked to create interactive activities on the basis of the enterprises. The reason for choosing 56 participants is that all kinds of personnel are distributed in different occupational types and roles, and the number allows understanding the attitude of different people toward AI technology from many aspects. At the same time, professional practitioners are avoided, who can better observe and analyze the AI technology attitude of the object in value co-creation, as we were seeking the perspective of ordinary consumers and participants. Their Occupational Roles are distributed in government, enterprise management, professional and technical personnel, students, and freelancers, among others. To integrate AI technology and application, the study focused on combining key points and requirements for each point of view. A total of 128 statements were obtained, and these 128 statements were regarded as propositions. Afterward, 104 propositions were deleted, and 24 valid propositions were retained in consideration of similarity, coincidence, and validity, which were used as Q sample propositions. Looking at various propositions, consumers’ concerns are about AI technology focused on multiple dimensions, such as interaction, recommendation, decision making, and application. Tables 1, 2 present the demographic information and research context.

**TABLE 1:** Basic description of participants.

Gender	Age
Male: *N* = 26	25–30 years old: *N* = 12
Female: *N* = 30	31–40 years old: *N* = 23
**Income level**	41–50 years old: *N* = 14
¥4,001–6,000: *N* = 12	51 and older: *N* = 7
¥ 6,001–8,000: *N* = 19	**Degree of education**
¥8,001–10,000: *N* = 15	College: *N* = 16
¥10,001 or more: *N* = 10	Bachelor: *N* = 29
	Master/Doctor: *N* = 11
	**Occupations roles**
	Government: *N* = 8
	Enterprise:*N* = 9
	Professional(doctor/lawyer/The teacher, etc.): *N* = 9
	Freelancer: *N* = 7
	Students: *N* = 9
	Business services (Sales person/The waiter): *N* = 9
	Agriculture, forestry, animal husbandry, and fishery workers: *N* = 5

**TABLE 2:** List of subjective statements (propositions).

Serial number	Subjective statement (propositions)
1	Openness and transparency of data information
2	The content of the true real situation
3	Variety of functions
4	Personal information, data privacy, and security
5	Personal freedom of information choices
6	The readability of content
7	Acceptance and feedback of self-true opinions
8	Contribute to the promotion of purchasing decisions
9	Can prompt what I didn’t expect
10	I can control different levels of artificial intelligence services
11	Can achieve two-way interaction with the provider
12	Can effectively solve the purchase problem I encountered
13	Make me feel real and anthropomorphic
14	Can make me feel trustworthy
15	Low risk of service provision
16	The content of the message is what I expect
17	Accuracy of information content
18	Able to respond quickly to my needs
19	Can bring me a sense of technology
20	More efficient than doing it myself
21	Personal views and opinions can be followed
22	Can bring me novelty and fun
23	Can make me feel involved
24	Can help me find low-price and high-quality goods or services

Second is the P sample measurement stage, which refers to the screening of test subjects related to the research topic to form a P sample set. The advantage of the Q method is that small samples can be adapted to measure cognitive attitude. Therefore, as long as the number of P samples is more than 30, the sample can be representative. The sample is randomly selected by using the survey sample database resources of a third-party market research company. The sample selection criteria are consumers who must have AI experience and have certain interaction experiences (online or offline) with commodity and service providers. The number of eligible samples is 42. Then, each test participant was asked to score the 24 propositions one by one according to his/her actual situation. The score was from a five-level scale (−2, 2), from strongly disagree (−2 points) to strongly agree (2 points). The score obtained supported the later Q classification stage.

**FIGURE 1:**
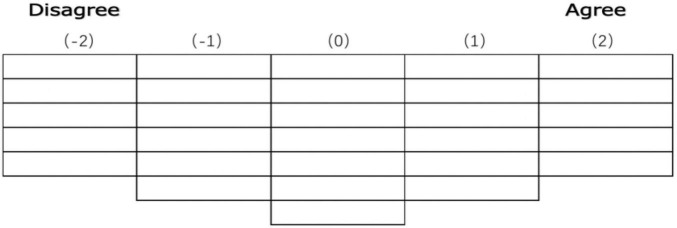
Five-level distribution table.

Third is the Q classification stage. After 42 people scored the propositions one by one, combined with the total number of propositions in this study, a five-level distribution table was specially established (Figure 1). The proposition scores of each tester were filled into the five-level distribution table one by one for subsequent analysis. After completing the distribution table, the researcher needs to conduct an in-depth interview on the extreme score items (strongly disagree, strongly agree) filled in by the test subjects. The researcher also needs to explore the reasons for the extreme score to understand the motivation and reason.

### Data Analysis and Interpretation

After data collection, PCQ Software is used to input and analyze the data. The analysis stage can be divided into three stages: factor analysis and rotation, factor load standard screening, and factor score and classification. The factor analysis and rotation stage is used for the definition and classification of topics, that is, how many categories of topics (factors) can the 24 proposition items be divided into? The factor load standard screening stage is used to screen specific propositions under each theme, and the propositions with low representativeness are deleted. The factor scoring and classification stage is to sort representative propositions with high weight in the case of clear subject classification and proposition items. Through the analysis of the three stages, consumers’ demands and attitudes toward the application of AI in VCC are focused on what topics. Which proposition items are the most important to consumers? Here, three stages are analyzed one by one.

### Factor Analysis and Rotation

The correlation matrix and eigenvalue were calculated for the 42 sample data. The samples fell into the first four factors, and six samples were excluded. The effective rate of samples falling into the factors was 85.7% (>80%, boundary standard), indicating that the four factors had been representative. Therefore, we can conclude four types of themes (factors) that consumers focus on (Table 3).

**TABLE 3:** The number of factors and samples that fall on the factors after rotation.

Explanatory variables(factor)	Explanatory sample ratio (%)	The number of propositions that fall on the factor
F1	21.3	6
F2	15.3	4
F3	28.6	6
F4	23.2	6
total	88.4	22

### Factor Load Standard Screening

In the Q method, for the factor loading to classify the sample for Q (subjective statement), the formula is expressed as: load > 4 (Number of factors)/$\left\lceil \sqrt{n}\right\rceil$ (*n* is the number of Q samples, and the number in this study is 24). The computational load factor is greater than 0.4908 as classification criteria were divided into different categories. In this stage, two non-conforming propositions were deleted, and the remaining 22 propositions were assigned to four different categories of topics (factors).

### Factor Score and Classification Results

After factor analysis and rotation, the number of topics (factors) and factor load value are determined to classify Q samples into factors. The proposition items that do not meet the standard are deleted. Afterward, the factor score was calculated for the Q sample. Its purpose is to clarify the key proposition items under each theme (factor), which has become the focus of consumers’ attention and needs deep observation and explanation (Table 4).

**TABLE 4:** Q sample classification and factor scores.

Factor 1 (Content quality)			Factor 2 Information security)			Factor 3 (Efficiency concern)			Factor 4 (Degree of manipulation)		
Serial number	statement	score	Serial number	statement	score	Serial number	statement	score	Serial number	statement	score
16	The content of the message is what I expected	1.836	14	It makes me feel trusted	1.962	12	Can effectively solve my purchase problem	1.653	23	It makes me feel involved	1.845
2	The true state of the content	1.574	4	Personal information, data privacy and security	1.424	20	More efficient than I could do it myself	1.542	10	Can control different degrees of artificial intelligence services	1.456
17	Accuracy of information content	1.345	1	The degree of openness and transparency of data information	0.935	8	Facilitate decision making	1.234	11	You can have two-way interaction with the provider	1.232
6	The accuracy of the content of the message	1.112	15	Low risk of service delivery	0.342	3	Features offer diversity	0.815	9	I can tell you what I didn’t expect	0.673
22	Can bring me novelty and interesting	0.946				18	Able to respond quickly to the needs I put forward	−0.244	21	Personal views and opinions can be taken into account	−0.065
19	It gives me a sense of technology	−0.245				24	It helps me find good quality goods or services at low prices	−0.513	13	It makes me feel real and personified	−0.134

## Findings

Through factor analysis of subjective propositions according to the Q method, four kinds of themes (factors) are obtained: “companies–consumers” in the process of value creation consumer AI techniques used for the proposed concerns can be divided into factor 1 (content quality), factor 2 (information security), factor 3 (efficiency concern), and factor 4 (degree of manipulation).

### Interpretation of Content Quality Factors

As can be seen from Table 4, in the process of VCC with enterprises, consumers have high concerns and expectations for the accuracy (1.836), authenticity (1.574), and accuracy (1.345) of information content, which is an important foothold for AI technology to connect the information and content interaction between “enterprises and consumers.” The current AI technology makes the information flow between “business and consumer” smooth, further affecting the consumer’s feedback to the business side of the information. Machine learning in AI technology can mine the behavior rules of consumers to realize accurate marketing and relevant recommendation of goods and services, which largely satisfies the statement that “information content is what I expect.” In the whole process of VCC, accurate and effective information interaction and transmission will be related to the further optimization and iteration of products and services of both parties and then affect the realization of the core value proposition.

Moreover, notably, consumers do not score high for the statement “can bring me a sense of science and technology.” A deep visit to consumers found that:

*“I don’t know any technology, I don’t follow any fashion. As for the high-end or not, I have no special feeling. This kind of technical things, I think it is not necessary to make so fancy, simple point, all kinds of advertising, information, introduction, activities, what can be understood at once on the line”—interview object (VCC2021032309)*

In view of this, when “business–consumer” uses AI to interact with information content, the readability of information and the ease of use of technology should be focused on. Excessive use of technology will hinder information transmission and feedback.

### Interpretation of Information Security Factors

Based on data with the aid of the application of AI for behavior data collection and use, such as computer vision personal image scanning (pay), the sound and voice recognition (acoustic equipment), biometric personal physiological indexes collection line (stores), and others, is not efficient for customers. These technologies directly touch data acquisition and personal privacy. The perspective of its collection channels and scale is cross-platform and ubiquitous. The collection and use of this series of information help to draw a complete and clear portrait of consumers, and enterprises can carry out various co-creation activities based on the portrait.

Among the information security factors, the statement of information privacy (1.424) and transparency (0.935) mentioned above are of high concern. However, the statement in first place is “can make me feel trust” (1.962), which indicates that with the popularization of informatization, consumers do not blindly emphasize the exclusion of their information sharing. Sharing depends on information given about the business, technology, and credibility concerns. For instance, the enterprise itself has a good sense of trust and effective protection for the information, which can be usedto promote the consumer to the sharing of information to dispel the concerns about potential risks, and for the enterprise to become closer to the consumer, and this trust can be built up.

*“Personal information is so common now that I have lost track of how much information I have put in. There’s nothing new about this, except for me, the main concern is who asked me for this information? Which enterprise, the company asks me to want, as long as I believe of, I will give, also want to have a reason of course.”—Interviewee (VCC2021031204)*

### Efficiency Concerns the Interpretation of Factors

Among the four types of factors, the highest efficiency concerned factor explanation has a higher weight. To a larger extent, this factor reflects the consumers for the application of AI technology, which can trigger efficiency. The remaining factors are the top three statements for problem-solving (1.653), more efficient than (1.543), and the decision of promoting (1.234). In the past VCC between “enterprises and consumers,” corresponding efficiency driving tools is limited. The application of AI technology aims to solve the matching efficiency problem between them, which further accelerates the realization of VCC.

Based on the concerns of consumers, AI technology mainly focuses on problem-solving and decision support. Current companies began to layout the robot process automation technology. Customer service, to use, is implemented in the efficiency of problem-solving optimization. Consumers can be provided for through the enterprise process automation, solving many common problems, improving efficiency, and promoting satisfaction and loyalty. Moreover, enterprises adopt various recommendation algorithms, such as group-based collaborative filtering and individual interest recommendation algorithms. This process, to a large extent, saves consumers’ time searching, evaluating, and selecting products or services, reduces their transaction costs, and achieves assistance for consumers’ decision-making to a certain extent.

*“I’m so busy at work every day that I just want to relax and I don’t have the energy to focus on solving one problem or another. I just want people to tell me how to solve this problem? What’s better? What can I do to achieve… The faster and easier it is, the better. Whoever can help me solve it the quickest, I feel the best.”—Interviewee (VCC20210420021)*

### Interpretation of the Degree of Manipulation Factor

The so-called degree of control reflects the degree of consumers’ dominant control in the co-creation process of “enterprise and consumer.” Among relevant statements, the sense of participation (1.845), controllability (1.456), and two-way interaction (1.232) rank the top three. Thus, consumers have a strong demand for participation in co-creation. For consumers, the application of AI technology should have the option of being selected rather than a single passive use. The AI-enabled link between business and consumer needs to be bi-directional, which is different from the one-dimensional connection between businesses and their consumers. Consumers are no longer passive subjects, and there is a switch to the active appeal. From the perspective of the manipulation degree factor, more emphasis is placed on the institutional issues triggered by AI, such as participation demand, initiative demand, and two-way interaction demand, thereby constructing the basic elements of VCC.

*“I want to have a chance to interact and express myself,” he said. “The technology is so advanced that I sometimes get involved and express my ideas. Some people text me back, but I don’t know if it’s a human or a robot, but I find it interesting.”—Interviewee (VCC2021040911)*

Moreover, the results show that the score of “makes me feel real and personified” (−0.134) is not high, and consumers’ demand for the “quasi-human” construction realized by AI is not so strong. The key behind it still needs to return to the essence of AI technology: the core debate of whether to assist humans or replace humans. For now, consumers do not show a particularly strong interest in “quasi-human” images.

## Discussion

The four kinds of factors have become the key to influencing VCC, but each has its own differences and emphases. According to the size of the interpretation rate, consumers’ concerns and weight for the factors were determined. In order of importance from high to low, the elements were divided into efficiency concern, control degree, content quality, and information security factors. The efficiency concern factor reflects consumers’ concern for efficiency, which focuses on problem-solving and decision assistance. Enterprises need to pay special attention to the selection and application of AI technology and realize the efficiency improvement of the VCC process by improving the efficiency of core demands. The degree of manipulation factor explains consumers’ concern about participation, interaction, and choice rights. The application of AI technology is not only one-dimensional but also needs to highlight the technological empowerment of choice and interaction. Content quality factor emphasizes the transmission of accurate information, and the authenticity and accuracy of content are the focus of consumers’ attention. In the process of co-creation, enterprises need to constantly use technology to explore the behavior rules and needs of consumers, to guarantee the quality of VCC. Information security factors highlight the importance of enterprise trust cultivation, and enterprises need to strengthen their own brand influence, reputation, and other aspects of the shaping. AI technology is trying to collect all kinds of information from consumers. By reducing consumers’ concerns about information privacy and security, we can truly encourage consumers to share effective information, which plays a key role in enterprises’ access to consumers.

## Implication

The combination of human and non-human behaviors has become a new mode of VCC. The traditional VCC is often the direct interaction between “enterprise and consumer” or multiple subjects. With the integration of AI technology, more non-human subjects and behaviors are added in the process of VCC. According to the ANT theory proposed by Bruno Latour, in a specific action system, actors are jointly realized by human and non-human behaviors. Moreover, non-human behaviors represent abstract products, such as technology, information, and institutions (Latour, 2005). Therefore, in the action system of VCC, the emergence of non-human actors, such as AI, adds many new features, new forms, and new contents to the traditional VCC. This emergence will further affect the form of VCC and make it become a value co-innovation mode under the joint action of human and non-human actors. In this mode, the past co-creation of enterprises and consumers may be replaced or enhanced to make it more efficient and achievable. Moreover, such co-creation can bring a series of potential problems, however, to create value in the AI technology and has begun to show its new vitality. This case is driven by consumers for the cognitive attitude and is gradually inclined to clear. This model has important implications for the enterprise and also for value to create relevant theory research and provides a possible future research path. This topic is worth deepening and exploring in the next stage.

Based on the high-weight proposition item of this study, consumers focus on the precision, information security, and interactivity of AI in VCC. In terms of efficiency, enterprises can further improve the computing power and algorithm of AI and continuously improve the accuracy of all kinds of information transmission through continuous training of consumers’ behavior and attitude data. This case will be directly reflected in specific behaviors, such as recommendation, evaluation, and feedback in co-creation activities. In terms of information security, network security technology requires further strengthening to avoid non-subjective information leakage. Daily management, such as data desensitization storage and information personnel management, can also be enhanced, which is the key to promoting consumers’ deep participation in co-creation activities. In interactive ways, enterprises can expand the AI that can give interactive scenarios, such as intelligent customer service, virtual community, VR images, and human-computer interaction, through the richness of interactive scenes and interactive forms, promote the participation of consumers to create activities, maintain its continuous, convenient interaction between businesses, and improve the quality of value creation.

The form and content of value co-creation between “enterprise-consumer” will become more different and diverse with the application of AI. For enterprises, selecting AI technology in a targeted manner is necessary according to the characteristics of their own products and services to avoid overuse or underuse. Starting from the factors that consumers are concerned about, the application of AI technology in a targeted manner can promote the achievement of co-creation performance, not only mastering AI technology but also matching it to consumer expectations. For consumers, they can learn relevant AI application technologies to improve the efficiency of interaction and communication between themselves and enterprises to better enhance their service experience. At the same time, with a relatively open mind, in face of the addition of new technologies and new methods, they can continuously promote their own immersion and use AI technology with enterprises to achieve new practices of value co-creation.

## Limitation of Recent Studies

A summary of relevant research shows the following characteristics. First, in terms of research objects, enterprises, consumers, and stakeholders should still be the main action subjects. However, few studies on non-human actors as the main body of AI still exist, though they are increasingly becoming the focus. Second, in element excavation, the traditional influence factors of VCC are founded on the basis of traditional scenes. However, with the embedding of AI technology, co-creation behavior becomes increasingly complex. The applicability of its influence factors in the new environment and the emergence of new elements need further verification and exploration. Finally, in terms of research methods, most of the previous studies were based on case studies, structural equations, and theoretical deduction. They lacked diversity in methods, which limited the deeper exploration of VCC behavior. To sum up, problems in previous relevant studies focus on the limitations of research objects, adaptability of elements, and innovation of research methods. This study aims at the above limitations, which are supplemented and improved.

## Future Research Agenda

Judging from the ranking of the four types of factors, the efficiency concernsof consumers has become the primary factor. The application focus of AI should not only be limited to the advancement of technology but also the quality of problem-solving and communication and other aspects of the pursuit of efficiency. In addition, consumers’ attention to the factor of the degree of manipulation reflects their emphasis on autonomy in the process of AI application. They can actively control the application of AI instead of passively accepting AI technology. These two points have become a special concern of consumption and become the core points of “enterprise-consumer” value co-creation, which requires special attention and grasp.

The positive significance of AI for VCC slowly allows it to become a research hotspot in the next stage and a new perspective apart from enterprises and consumers. Future research directions can be deepened from the following aspects. First is in-depth research on specific industries. At present, research on VCC remains in the stage of theoretical deduction and concept construction and is not deeply embedded in specific industries, and needs further strengthening. Different industries have different degrees of AI application. Key research can be carried out by combining industries with a higher degree of AI application, such as retail, logistics, and the Internet, to discuss the specific application of AI in VCC. These industries can reflect that AI plays a key role in co-creation between enterprises and consumers.

Second is the use of AI in social media. Social media has gradually become an important practice place for VCC between “enterprises and consumers,” which is also the key scene that AI can embed, such as intelligent recommendation, evaluation feedback, intelligent customer service, and others, which help VCC. The research on its occurrence and operation mechanisms will also be meaningful. It can help enterprises improve their daily operation.

Third is the double-sided effect analysis of AI in VCC. Past research in a positive perspective on AI interventional effect focused less on the AI for value to create a negative impact, specifically the AI, which destroyed the possibility of research for value. The value for related research has been divided; its enthusiasm is no longer discussed separately. Moreover, a theory about the negative effect is increasing, including AI and value creation, by combining the theme of the debate. As for the research on value co-destruction, only the real environment is the focus, whereas the virtual environment is ignored. However, in many cases, AI applications are carried out in the real environment and not in the virtual one. Therefore, exploring the value co-destruction of AI in the virtual environment will have pioneering research significance.

Fourth, we evaluate the differential effect of AI technology on VCC. Many existing technologies in AI exist, such as machine learning, neural networks, and natural language. Different technologies have different functions and values and have different attractions to enterprises and customers. They can start with the most frequently used AI technology, explore its practical value and significance, evaluate which technologies will become the core technologies to promote VCC, and then carry out improvement and development.

## Data Availability Statement

The original contributions presented in the study are included in the article/supplementary material, further inquiries can be directed to the corresponding author.

## Ethics Statement

Ethical approval was obtained from the Shanghai Jianqiao University before carrying out the survey. The patients/participants provided their written informed consent to participate in this study.

## Author Contributions

YZ: overall planning of the full text, selected methods, and analysis of the conclusion. PW: collect data, process data, and correct text. WD: revising and organizing the full text of the manuscript, as well as taking care of the later revisions of the manuscript. All authors contributed to the article and approved the submitted version.

## Conflict of Interest

The authors declare that the research was conducted in the absence of any commercial or financial relationships that could be construed as a potential conflict of interest.

## Publisher’s Note

All claims expressed in this article are solely those of the authors and do not necessarily represent those of their affiliated organizations, or those of the publisher, the editors and the reviewers. Any product that may be evaluated in this article, or claim that may be made by its manufacturer, is not guaranteed or endorsed by the publisher.
